# Frontiers in Retinal Image Processing

**DOI:** 10.3390/jimaging8100265

**Published:** 2022-09-29

**Authors:** Vasudevan Lakshminarayanan, P. Jidesh

**Affiliations:** 1 Theoretical and Experimental Epistemology Lab, School of Optometry and Vision Science, University of Waterloo, Waterloo, ON N2L3G1, Canada; 2 Department of Mathematical and Computational Sciences, National Institute of Technology Karnataka, Surathkal 575 025, Karnataka, India

Visual impairment is considered as a primary global challenge in the present era. This is due to numerous reasons such as lack of awareness, shortage of resources and trained personnel, inability to seek immediate medical treatments, etc. The major modalities for non-invasively examining the retina includes procedures such as fundus photography, and optical coherence tomography (OCT). Another popular modality is fluorescein angiography. These images are subject to expert analysis for further diagnosis. However, due to the extent of clinical observations needed on a day-to-day basis, it is not practically viable to manually analyse these data.

Automated retinal image analysis has therefore turned out to be an inevitable processing technique in modern retinal analysis. The automated analysis can assist in diagnosing the disease and grading them as well as monitoring the progression or regression of the disorder. Glaucoma, age-related macular degeneration, macular edema, and diabetic retinopathy are some of the widespread retinal disorders that are progressive and require frequent follow-ups. State-of-the art devices such as portable OCT, smart-phone camera attachments, etc., have simplified the acquisition of retinal images to some extent. Nevertheless, the ever-increasing blind population and the availability of massive computational resources have spurred the urgent need to develop automated retinal imaging applications. The gamut of cutting-edge technologies such as artificial intelligence and deep learning is a possible gateway to address these challenges. This Special Issue mainly focus on these issues and challenges. 

This Special Issue received several submissions. All submitted papers underwent a rigorous peer-review process. After thorough revisions, five articles were selected based on the expert’s opinion. 

In the paper titled “Intraretinal Layer Segmentation Using Cascaded Compressed U-Nets” [1], the authors propose and validate a cascaded two-stage network for intraretinal layer segmentation, with both networks being compressed versions of U-Net (CCU-INSEG). The first network in this model is responsible for retinal tissue segmentation from OCT B-scans. Further, the second network segments eight intraretinal layers with high fidelity. The model is tested and validated using various datasets.

In the paper titled “Unsupervised Approaches for the Segmentation of Dry ARMD Lesions in Eye Fundus cSLO Images” [2], the authors propose an adaptation of a fully convolutional network, called W-net, as an efficient method for the segmentation of ARMD lesions. The model is tested on a large dataset and the results are shown to be promising.

In the paper titled “EffUnet-SpaGen: An Efficient and Spatial Generative Approach to Glaucoma Detection” [3], the authors develop a two-phase model for glaucoma detection, identifying and exploiting a redundancy in fundus image data relating particularly to the geometry. A novel algorithm is proposed for the cup and disc segmentation with an efficient convolution block and combines this with an extended spatial generative approach for geometry modelling and classification. The method is tested with a different dataset to demonstrate the performance of the model.

In the paper titled “Towards a Connected Mobile Cataract Screening System: A Future Approach” [4], the authors review the development and limitations of published methods for cataract detection and grading using different imaging modalities. Finally, they conclude that the use of digital images from a smartphone as the future of cataract screening tools could be a practical and helpful solution for ophthalmologists, especially in rural areas with limited healthcare facilities.

Finally, the review paper titled “Automated Detection and Diagnosis of Diabetic Retinopathy: A Comprehensive Survey” [5], covers the literature dealing with AI approaches to DR such as ML and DL in classification and segmentation that have been published in the open literature within six years (2016–2021). In addition, a comprehensive list of available DR datasets is reported. 
